# Treating a rare mid-term complication of transapical transcatheter aortic valve implantation (TAVI): a case report

**DOI:** 10.1080/23320885.2022.2070490

**Published:** 2022-05-13

**Authors:** Eugenio Fraccalanza, Alessandra Fin, Nicola Zingaretti, Filippo Contessi Negrini, Enzo Mazzaro, Ugolino Livi, Pier Camillo Parodi

**Affiliations:** a Plastic Surgery Unit, Azienda Ulss 3 Serenissima, Venezia-Mestre, Italy; b Hand Surgery and Reconstructive Microsurgery Unit, ASST G. Pini-CTO, Milan, Italy; c Clinic of Plastic and Reconstructive Surgery, Academic Hospital of Udine, Department of Medical Area (DAME), University of Udine, Udine, Italy; d Cardiac Surgery Service, Humanitas Gavazzeni Hospital of Bergamo, Bergamo, Italy; e Cardiac Surgery Service, Academic Hospital of Udine, Department of Medical Area (DAME), University of Udine, Udine, Italy

**Keywords:** Heart valve replacement, transapical, healing, dehiscence, wound closure, chestwall, perforator flap

## Abstract

We report the case of a patient who underwent transcatheter aortic valve implantation *via* left anterior mini-thoracotomy in whom a periapical subcutaneous collection appeared in the anterior thoracic wall 18 months after the procedure. The tissue defect was efficaciously repaired *via* intercostal artery perforator flap, preserving the thoracodorsal and internal mammary arteries.

## Case report

An 81-year-old male patient presented with diagnosis of severe aortic stenosis and a history of chronic ischemic cardiopathy with previous percutaneous transluminal coronary angioplasty (PTCA) and a coronary artery bypass grafting; recent vein graft PTCA for the posterolateral branch of the circumflex artery; carotidopathy and abdominal aortic aneurism with thrombotic stratification. There were symptoms of heart failure (NYHA II), and the Heart Team opted for TA-TAVI.

On November 2016, the patient underwent TAVI to implant a 3–29 mm Edwards Sapien valve (Edwards Lifesciences, Irvine, CA, USA) *via* left anterior mini-thoracotomy. Both the operation and the immediate post-operative course were devoid of significant complications, allowing rapid weaning off pharmacological support, and extubation after 6 h. After 6 days, the patient was discharged in good general clinical and cardiocirculatory condition. The surgical site was as expected at discharge and early post-operative check-ups.

However, roughly 18 months after surgery, and despite appearing in good health and totally asymptomatic, the patient was once more seen by the heart surgeon due to a previously unnoticed apex beat suggestive of left ventricular pseudoaneurism. Thoracic CT revealed a 40 × 54 × 39 mm plurilocular accumulation with density greater than fluid from the anterior wall of the left ventricle to the subcutaneous soft tissues, through the intercostal space between ribs 5 and 6; its walls were significantly enhanced after contrast. Cardiac MRI was indicative of siero-haematic accumulation with no evidence of communication with the left ventricular cavity or signs of active bleeding.

Surgery was performed to drain the accumulation, exposing the cardiac apex and removing the sutures and foreign matter from the previous operation (sent for culture, this was negative for bacterial growth). Curettage was performed, leaving the thoracotomy open until the wound was clean. Antibiotic therapy with Daptomycin was administered in the post-op and for 2 weeks. After three weeks there was a wide, full-thickness defect in the thoracic wall exposing left ventricle movement.

**Figure 1.**
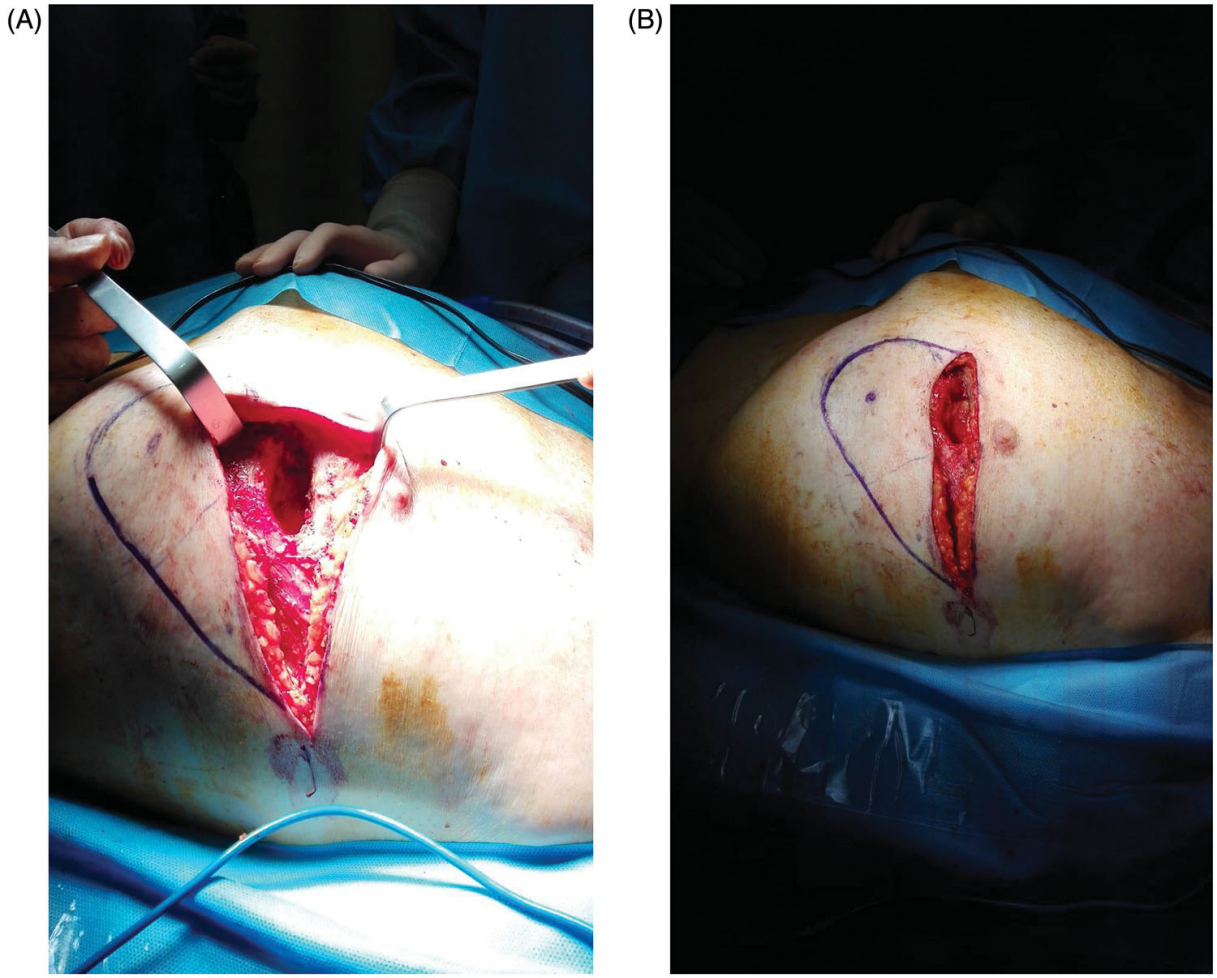
(A) The defect after debridement. (B) Flap design. The blue dot marks the perforator identified with the handled doppler probe.

**Figure 2.**
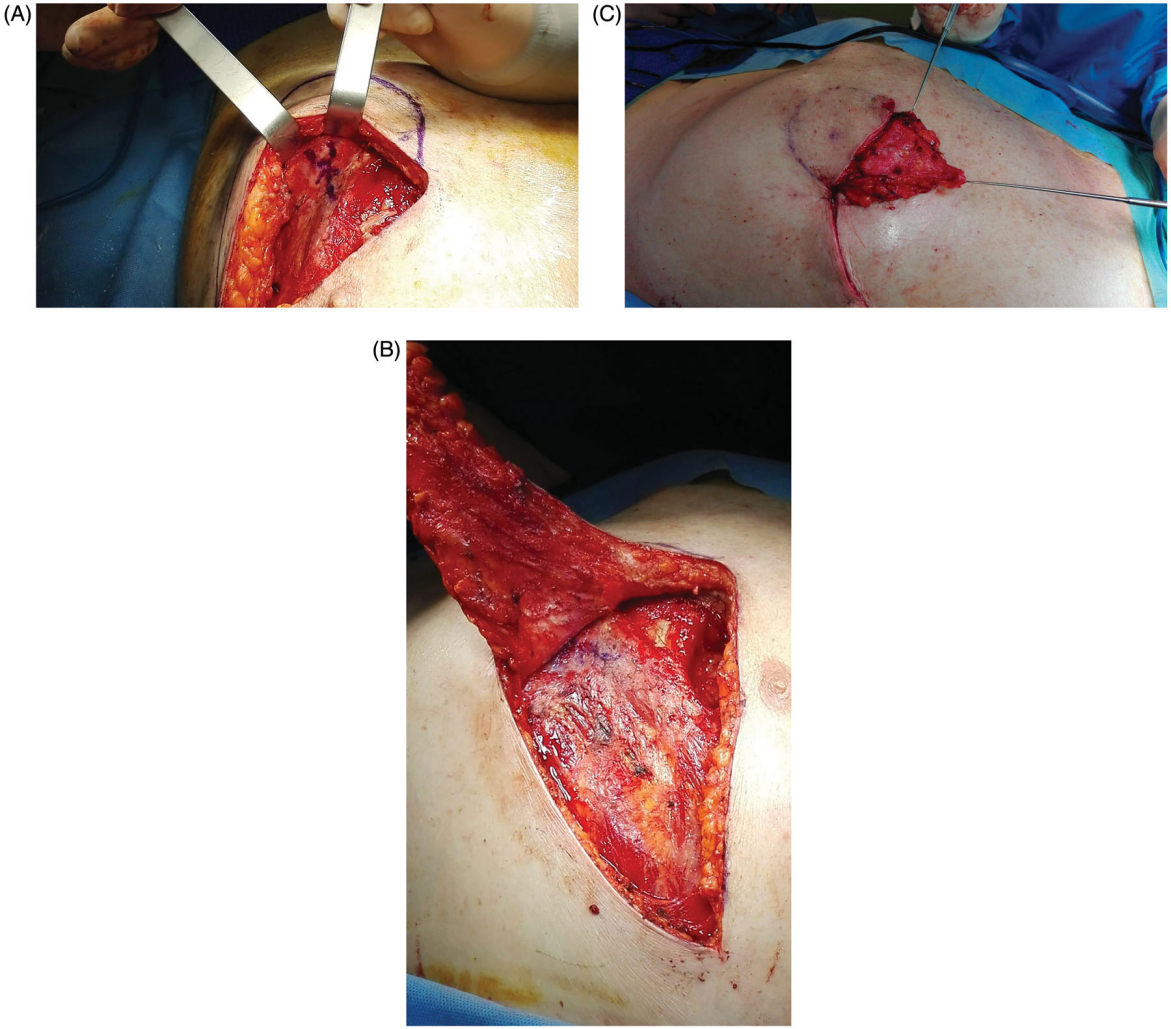
Flap raising. After perforator identification, incision of the lower margin and flap raising (A). (B) De-epithelialization and transposition of the proximal portion of the flap. (C) The wound 1 week post-op.

**Figure 3.**
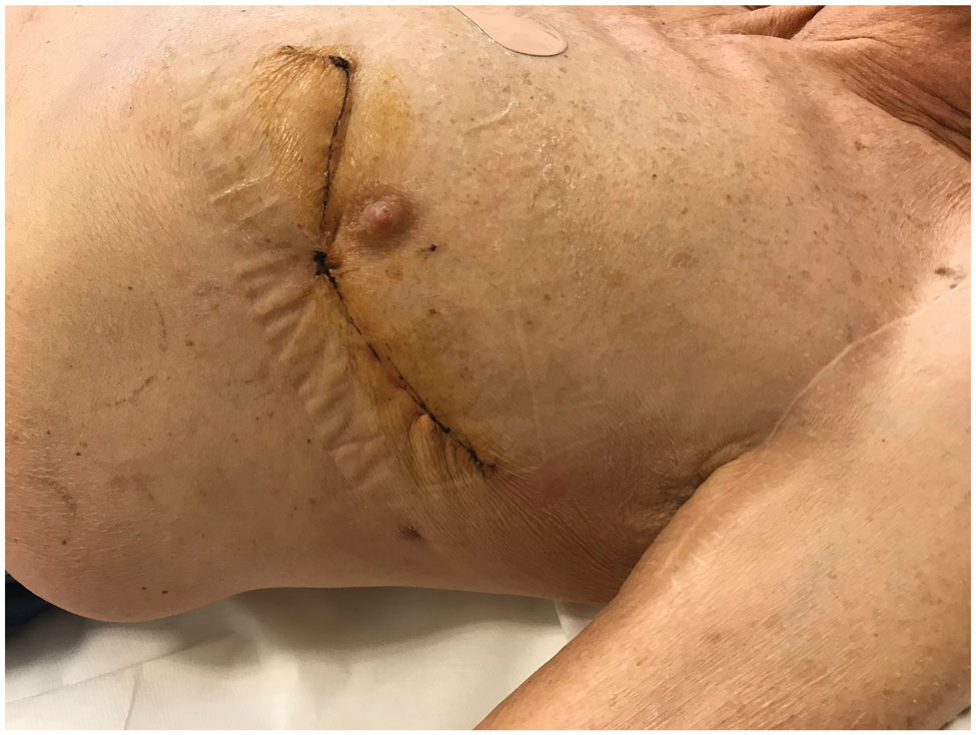
At one month post-op the flap was completely consolidated with the thoracic tissues, scars were well healed and no distortion of the thoracic surface was noted.

**Figure 4.**
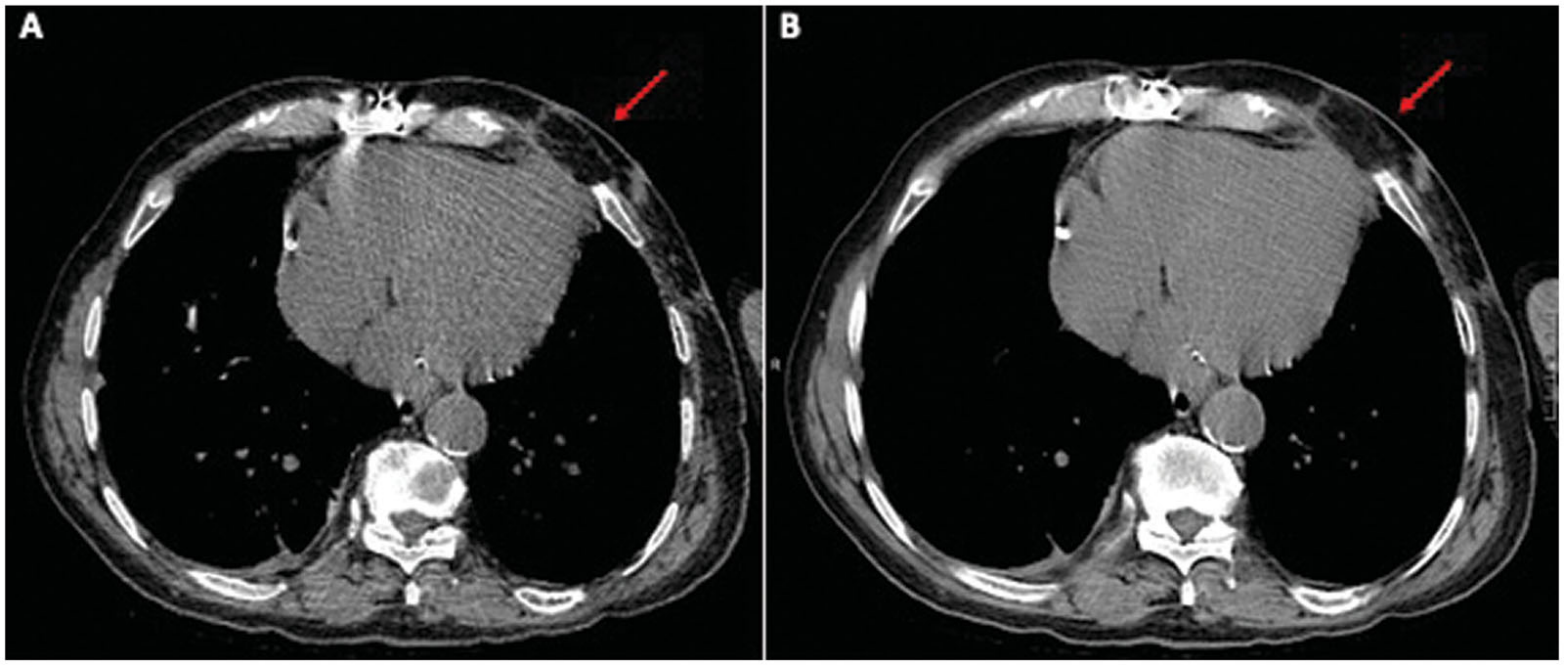
A CT scan executed 1 month post-op (A) and 1 year post-op (B) clearly shows the trophism of the flap (red arrow) and no collection.

The Plastic Surgery proposed *via* perforator flap repair. A portable Doppler probe was used to identify an intercostal artery perforator in the 6^th^ intercostal space [1]; a tear-shape flap was centred on this vessel, with the upper margin of the flap coinciding with the lower margin of the defect (Figure 1). After debridement, the flap was raised and the pedicle directly examinated. We found that the flap could be sufficiently mobilised without using the island technique, thereby sparing the inferior and medial extensions of the surgical incision. The portion of tissue distal to the perforator was de-epithelialized and used to fill the defect; fibrin sealant Tisseel (Baxter Healthcare Corp., Deerfield, IL) was applied to promote flap adhesion to the surrounding tissue and further reduce dead space (Figure 2(A–C)). The wound was closed by bringing the upper margin of the flap to the upper margin of the defect (Figure 3). Surgery time for wound cleaning and reconstruction was 100 min. At the last clinical and CT control (June 2019) the flap was completely consolidated with the thoracic tissues, scars were well healed and no distortion of the thoracic surface was noted (Figure 4(A,B)).

## Discussion

In diseased aortic valves, Transcatheter aortic valve implantation (TAVI) is a minimally invasive alternative involving implanting an expandable prosthesis inside the native valve. First used in patients with prohibitive or high surgical risk, its indications are expanding rapidly [2]. Various access routes for the procedure have been proposed [3], but in roughly 5% of cases [4], the transfemoral percutaneous route may be precluded. One of the most commonly used alternative implantation techniques is transapical TAVI (TA-TAVI), which is linked to fewer vascular complications, can be performed in patients with severe peripheral vascular disease or arteriosclerosis, and does not involve surgical manipulation of the aorta. Furthermore, the antegrade route enables more precise control of the device [5]. That being said, mini- thoracotomy at the cardiac apex exposes the patient to the risk of several complications at the access site.

Although nowadays considerably more percutaneous approaches are performed, TA-TAVI is still indicated in some cases, specifically severe vascular disease (in which the retrograde transfemoral approach is contraindicated) [6]. The increased mortality in TA-TAVI as compared to TF-TAVI is linked to the patient risk profile and surgical team experience rather than the procedure itself. Indeed, such patients generally have a higher pre-procedural risk (ASA and EuroScore II) and greater vascular system compromise, reducing the chance of rapid, efficacious healing of complex thoracic wall defects. In fact, according to some reports, the complications linked to TA mini-thoracotomy, albeit mainly acute and linked to direct access to the left ventricular cavity, may affect up to 8% of treated patients [7]. These include surgical site infection, ventricular pseudoaneurisms, and accumulations often secondary to slowly progressing infections, promoted by the presence of suture material at the left ventricular apex.

Wound dehiscence after cardiac surgery can represents a life-threatening condition. Patients suffering this complication should be promptly evaluated by an expert plastic surgeon. First of all, the present of soft tissue or bone infection should be taken into account, and an antibiotic therapy set up. Then a patient specific reconstructive project can be considered. Different are the possibility to repair the mid-thoracic wound such as negative-pressure wound therapy with continuous instillation (NPWT-CI) [8], secondary intention wound healing and loco-regional or free flaps.

Compared to secondary intention healing, flaps allow to faster repair the defect with a thicker, more elastic, more stable, and higher vascularized tissue.

Various flaps are described in published reports to reconstruct chest wall defects such as the pectoralis major flap [9], vertical rectus abdominis muscle (VRAM) or transverse rectus abdominis muscle flap (TRAM), greater omental flap, or latissimus dorsi flap [10,11].

Unless there are large voids or ribcage osteomyelitis, where a larger muscular flap or omentum flap are preferred, defects in the thoracic wall can easily be corrected with perforator-flaps based on the perforator branches of the major arteries that supply the anterior thorax (i.e. the internal mammary, the superior epigastric and the anterior and posterior intercostal). Different flaps can be designed on this perforator vessels with different shapes, sizes, orientations and positions; in an area from the second intercostal space to a few centimeters below the xiphoid process.

Such flaps are pliable and muscle-sparing, made up of skin and subcutaneous tissue; there are no functional sequelae, and major skin detachment is not required. They also spare the vascular pedicle of the thoracodorsal and internal mammary, which can be preserved for by- pass or other flaps, if necessary. Moreover, during surgery there is no need to change patient position, and donor site scarring is minimal. Indeed, local flaps can be rapidly and definitively closed without excessive burden on the patient and can be performed in the same session as surgical toilet without unduly extending operating time.

## Conclusion

Mid-thoracic wound dehiscence after cardiac surgery can represent a life-threatening condition. Patient suffering this complication should be promptly evaluated by an expert Plastic Surgeon who will manage the reconstructive project. Different are the possibility to repair these defects: from negative-pressure wound therapy with continuous instillation to free flaps. When there are not massive tissue voids or ribcage osteomyelitis, perforator-flaps based on the perforator branches of the major thoracic arteries (i.e. IAP flap) should be considered.

## Informed consent statement

The study was conducted according to the guidelines of the Declaration of Helsinki. Informed consent was obtained from the subject involved in the study. Written informed consent has been obtained from the patient to publish this paper.
